# The ratio of trichomes to stomata is associated with water use efficiency in *Solanum lycopersicum* (tomato)

**DOI:** 10.1111/tpj.14055

**Published:** 2018-09-05

**Authors:** Javier Galdon‐Armero, Mateu Fullana‐Pericas, Pere A. Mulet, Miquel A. Conesa, Cathie Martin, Jeroni Galmes

**Affiliations:** ^1^ Department of Metabolic Biology John Innes Centre Colney Lane Norwich NR4 7UH UK; ^2^ Research Group on Plant Biology under Mediterranean Conditions – INAGEA Universitat de les Illes Balears Carretera de Valldemossa km 7.5 07122 Palma Spain

**Keywords:** trichomes, stomata, water use efficiency, tomato, introgression lines, drought tolerance

## Abstract

Trichomes are specialised structures that originate from the aerial epidermis of plants, and play key roles in the interaction between the plant and the environment. In this study we investigated the trichome phenotypes of four lines selected from the *Solanum lycopersicum *× *Solanum pennellii* introgression line (IL) population for differences in trichome density, and their impact on plant performance under water‐deficit conditions. We performed comparative analyses at morphological and photosynthetic levels of plants grown under well‐watered (WW) and also under water‐deficit (WD) conditions in the field. Under WD conditions, we observed higher trichome density in ILs 11‐3 and 4‐1, and lower stomatal size in IL 4‐1 compared with plants grown under WW conditions. The intrinsic water use efficiency (WUE_i_) was higher under WD conditions in IL 11‐3, and the plant‐level water use efficiency (WUE_b_) was also higher in IL 11‐3 and in M82 for WD plants. The ratio of trichomes to stomata (*T*/*S*) was positively correlated with WUE_i_ and WUE_b_, indicating an important role for both trichomes and stomata in drought tolerance in tomato, and offering a promising way to select for improved water use efficiency of major crops.

## Abstract 2

Water availability is currently the biggest threat to agricultural productivity worldwide, and improving the water use efficiency of major crops has become essential. We analysed the epidermal features of near‐isogenic tomato lines and their response to drought, and concluded that the trichome‐to‐stomata ratio plays a major role in determining water use efficiency, thereby offering alternative paths for the agricultural improvement of tomato.

## Introduction

The epidermis is the outermost tissue of all plant organs and acts as a first contact point with their surroundings. It plays a key role in all plant–environment interactions, and is essential for the maintenance of physiologically favourable conditions (Glover *et al*., 2016). In the aerial organs of most terrestrial plants, including *Solanum lycopersicum* (tomato), the epidermis is patterned with trichomes, which are epidermal outgrowths with diverse roles in the defence against biotic and abiotic stresses. The epidermis also contains stomata, which are epidermal pores that regulate gas exchange and contribute directly to the control of water status. The cuticle that covers the surface of the epidermis is a hydrophobic layer, consisting of cutin and waxes, that prevents uncontrolled water loss (Riederer and Schreiber, 2001). As a result of their function in limiting water losses, specialised structures in the epidermis are promising targets to improve the drought tolerance and water use efficiency (WUE) of major crops (Antunes *et al*., 2012; Galmes *et al*., 2013; Franks *et al*., 2015).

Trichomes in *Solanum* are multicellular and have been classified into eight different types according to the presence or absence of glandular cells, and the shape and number of cells (Luckwill, 1943; McDowell *et al*., 2011). Research on trichomes has traditionally focused on understanding the specialised metabolic pathways operating in glandular trichomes (Schilmiller *et al*., 2008; Kang *et al*., 2014; Spyropoulou *et al*., 2014); however, trichomes also play a series of important physiological roles, including tolerance to biotic and abiotic stresses, especially in terms of tolerance to insect attack (Bleeker *et al*., 2012; Tian *et al*., 2012) and drought (Hauser, 2014).

A role for trichomes in tolerance and adaptation to water stress has been reported for several species. In *Arabidopsis lyrata*, trichome production has been linked to improved performance under drought conditions (Sletvold and Ågren, 2012). In tomato, SlMX1‐overexpressing plants with high trichome density showed a higher tolerance to water stress compared with unmodified plants (Ewas *et al*., 2016). In *Citrullus lanatus* (watermelon), wild, drought‐tolerant genotypes have increased trichome density compared with domesticated, drought‐sensitive varieties (Mo *et al*., 2016). In addition, trichome formation is increased in plants grown under water stress, such as *Hordeum vulgare* (barley; Liu and Liu, 2016), *Solanum melongena* (aubergine; Fu *et al*., 2013) and *Olea europaea* (olive; Boughalleb and Hajlaoui, 2011). Trichomes may limit water loss by transpiration through an increase of the leaf–air boundary layer resistance (Palliotti *et al*., 1994; Guerfel *et al*., 2009; Mo *et al*., 2016). Trichomes also protect leaves from UV‐related photoinhibition (Savé *et al*., 2000; Galmés *et al*., 2007a), either by reflection of UV radiation or absorption by pigmented molecules (Holmes and Keiller, 2002), and prevent leaf overheating (Ehleringer and Mooney, 1978). These findings indicate an important role for trichomes in plant–water relations.

Stomata consist of two specialised guard cells, which modulate their turgor to regulate the pore aperture in response to environmental stimuli, such as light intensity or CO_2_ concentration (Hetherington and Woodward, 2003). The effect of stomatal density and size on drought tolerance has been widely studied for many species (Masle *et al*., 2005; Wentworth *et al*., 2006; Lawson and Blatt, 2014). Recent studies have shown a link between low stomatal density and improved performance under water‐deficit conditions (Farber *et al*., 2016; Zhao *et al*., 2017). In addition, evidence exists that plants adjust their stomatal density under water‐stress conditions (Galmés *et al*., 2007b; Hamanishi *et al*., 2012). Reductions in stomatal density in water‐stressed plants have been reported in *Triticum aestivum* (wheat; Li *et al*., 2017) and *Spondias tuberosa* (the umbu tree; Silva *et al*., 2009). In contrast, for other species, stomatal density increases under drought conditions, as in *Phaseolus vulgaris* (the common bean; Gan *et al*., 2010) and *Leymus chinensis* (Xu and Zhou, 2008). These contradictory observations suggest that the effect of water deficit on stomatal density differs between species, and it should be investigated on a case‐by‐case basis.

*Solanum pennellii* is a drought‐tolerant wild tomato species that originates from the Peruvian deserts (Rick, 1973; Kahn *et al*., 1993; Peralta *et al*., 2008), with important differences at the physiological, morphological and molecular levels to the cultivated tomato, *S. lycopersicum*, including increased trichome density (Simmons and Gurr, 2005; Moyle, 2008). The *S. lycopersicum* × *S. pennellii* introgression line (IL) population (Eshed and Zamir, 1995; Zamir, 2001) consists of near‐isogenic lines with relatively small fragments of the *S. pennellii* genome in the genetic background of the cultivated tomato, M82. This population has been used successfully before to characterise various aspects of the response of tomato to water stress (Barrios‐Masias *et al*., 2014; Rigano *et al*., 2016). Therefore, the *S. lycopersicum* × *S. pennellii* IL population provides an excellent platform to investigate the role of differences in epidermal features on performance under water stress.

In this study, we have investigated the effect of differences in trichome density on the response to water stress using several lines from the IL population. We hypothesised that changes in trichome density, introgressed from drought‐tolerant *S. pennellii*, would lead to changes in WUE, adding to the current understanding of the relationship between water stress and epidermal features, and building a foundation for improvement of tomato under drought stress. We determined that higher trichome densities result in improved WUE, especially under water‐deficit conditions. We also determined the impact of water stress on the phenotype of newly developed tissues to characterise the epidermal responses to drought stress in tomato.

## Results

### Phenotypic characterisation of tomato lines under glasshouse and field conditions before drought treatment

After a preliminary visual inspection of the 76 lines of the IL population, we chose three ILs (IL 4‐1, 10‐2 and 11‐3) and the parental line M82, for their distinct trichome phenotypes, for further analysis.

**Figure 1:**
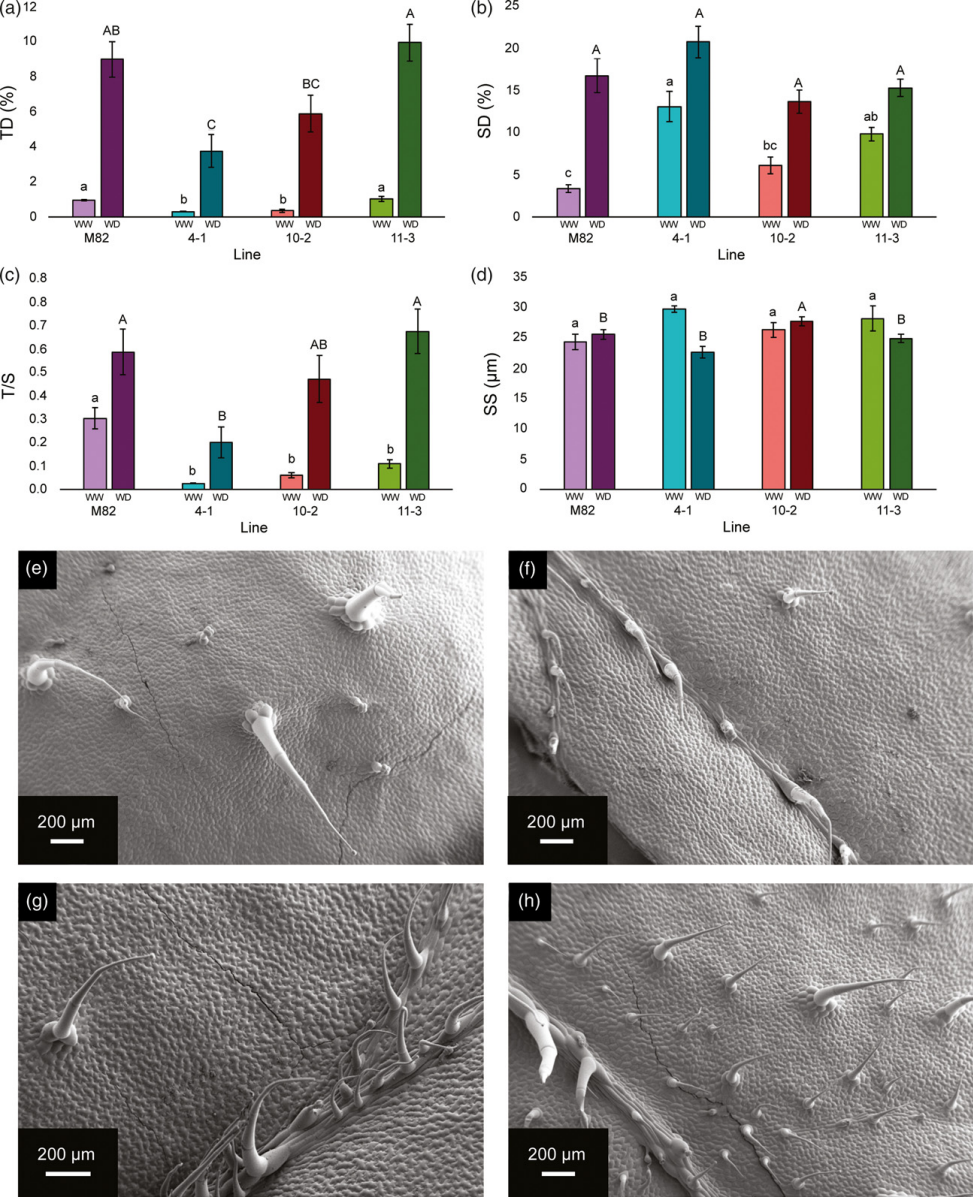
Initial morphological characterization of lines M82, 4‐1, 10‐2 and 11‐3 grown under glasshouse (GH) and field (F) conditions before the onset of drought treatment. (a) Trichome density (TD), (b) stomatal density (SD), (c) trichome‐to‐stomata ratio (*T*/*S*) and (d) stomatal size (*SS*) are expressed as mean ± SE of between three and six replicates per line and treatment. TD and SD were calculated as a percentage of the total number of epidermal cells. *SS* was calculated as pore length. Different letters denote statistically significant differences by Tukey's analysis (*P *< 0.05) within glasshouse‐grown plants (lower case) and field‐grown plants (upper case). For panels (a)–(d), purple bars represent M82, turquoise represents 4‐1, red represents 10‐2 and green represents 11‐3, with lighter and darker shades representing GH and F conditions, respectively. Representative scanning electron micrographs for each line: (e) M82, (f) 4‐1, (g) 10‐2 and (h) 11‐3.

We characterised the epidermal features of the three selected ILs (4‐1, 10‐2 and 11‐3) and the parental line M82 in plants grown under full field capacity conditions both in the glasshouse and in the field before the onset of water‐deficit conditions (Figure 1). Under glasshouse conditions, ILs 4‐1 and 10‐2 showed a low trichome density (TD) phenotype, whereas M82 and IL 11‐3 showed a high‐TD phenotype. Although field‐grown plants displayed substantially higher TD than glasshouse‐grown plants, IL 4‐1 had a lower TD than IL 11‐3 and M82, and IL 11‐3 had a higher TD than ILs 10‐2 and 4‐1 (Figure 1a). We also observed differences among lines in their stomatal densities (SD). Under glasshouse conditions, IL 4‐1 had a higher SD than M82 and IL 10‐2, and M82 had a lower SD than ILs 11‐3 and 4‐1. In field‐grown plants, no significant differences were observed between lines for SD (Figure 1b). Similar to the observations for TD, SD values were generally higher under field conditions. When TD and SD were calculated in terms of area units, we observed identical differences between lines for glasshouse‐grown plants, and similar relative values for field‐grown plants (Figure S1). We calculated the ratio of trichomes to stomata (*T*/*S*) as an integrative parameter for epidermal anatomy. We observed a substantially higher *T*/*S* in M82 compared with the other lines under glasshouse conditions, whereas in field plants, IL 4‐1 showed a lower *T*/*S* than M82 and IL 11‐3 (Figure 1c). Stomatal size (SS) showed no significant differences between lines under glasshouse conditions. Under field conditions, however, IL 10‐2 had a higher SS than the other lines under study. Unlike the higher values observed for TD and SD in field‐grown plants compared with glasshouse plants, the SS values were within a similar range under both growth conditions (Figure 1d).

We categorised trichomes into different types according to the established classification (Luckwill, 1943; McDowell *et al*., 2011) and measured the length of type‐I trichomes in glasshouse‐grown plants to determine whether there were differences in other trichome parameters between the ILs (Figure S2). We observed no significant differences in the percentage of different trichome types or trichome length. In general, the analysis of epidermal features and the comparison between glasshouse‐ and field‐grown plants indicated stable genetic components in the determination of trichome density.

### Characterisation of photosynthetic parameters under field conditions before drought treatment

We examined whether there were differences in parameters related to gas exchange, carbon fixation and RubisCO kinetics between the lines under study. We observed no significant differences in the net photosynthetic rate (*A*_N_), daily carbon fixation rate (*C*_24h_), stomatal conductance (*g*_s_), leaf mesophyll conductance (*g*_m_), leaf total conductance (*g*_tot_), maximum RubisCO carboxylation rate (*V*_cmax_) or intrinsic water use efficiency (WUE_i_) (Table S1), although we observed a trend to lower WUE_i_ values in IL 4‐1 compared with M82 (*P* < 0.06).

### Comparative characterization of epidermal features under well‐watered (WW) and water‐deficit (WD) conditions in the field

**Figure 2:**
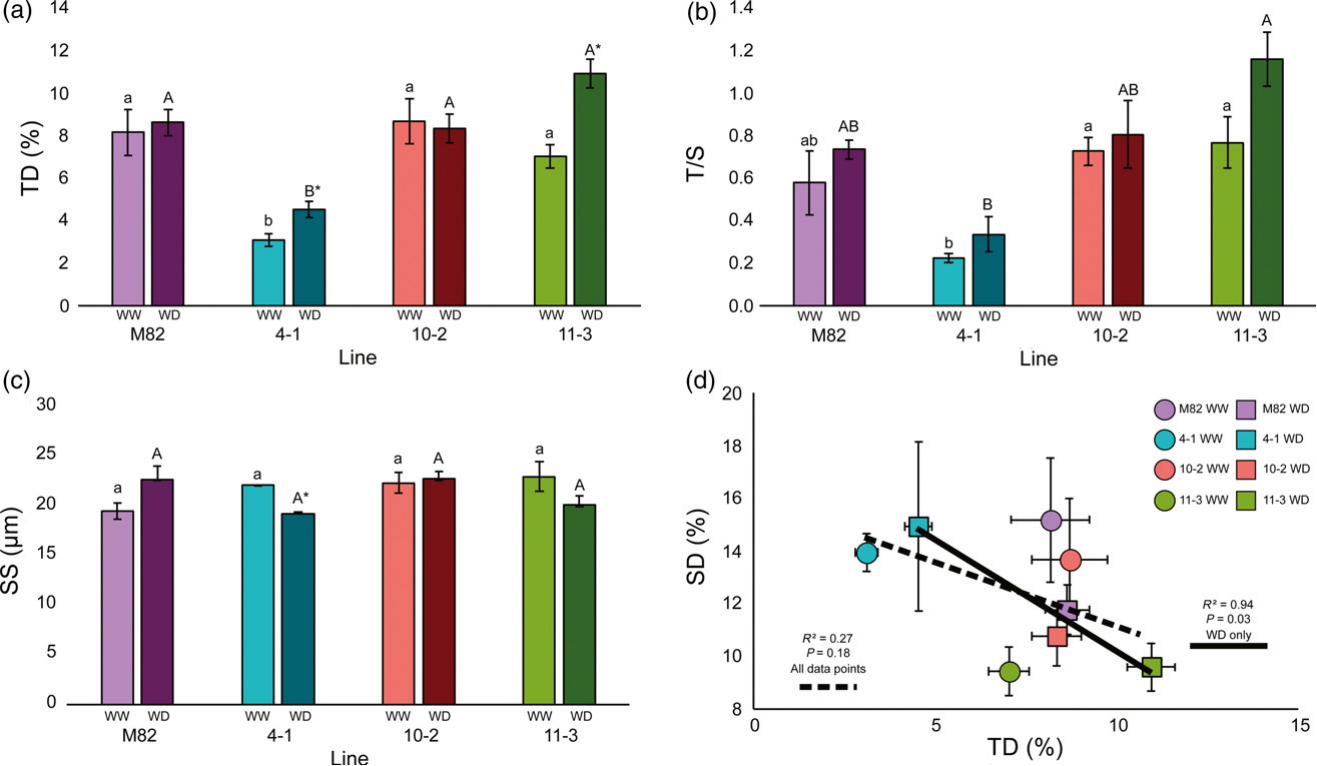
Characterization of epidermal features in lines M82, 4‐1, 10‐2 and 11‐3 under water‐deficit (WD) and well‐watered (WW) conditions in the field. (a) Trichome density (TD) is expressed as a percentage of the number of trichomes per epidermal cell. (b) Trichome‐to‐stomata ratio (*T*/*S*) is expressed as the total number of trichomes divided by the total number of stomata in a given area. (c) Stomatal size (*SS*) is expressed as pore length. Different letters denote statistically significant differences identified by Tukey's analysis (*P *< 0.05) within WW plants (lower case) and WD plants (upper case). Asterisks represent significant differences for each line between treatments according to the Tukey's test (*P *< 0.05). (d) A correlation between TD and SD was observed only under WD conditions, according to Pearson's test (*P *< 0.05), with the correlation index shown in the graph. For panels (a)–(c), purple bars represent M82, turquoise represents 4‐1, red represents 10‐2 and green represents 11‐3, with light and dark colours representing WW and WD treatments, respectively. In panel (d), squares represent WD values and circles represent WW values, and are colour coded as in panels (a)–(c). The dashed line represents the regression line for all data points (*P *> 0.05) and the solid line represents the regression line for WD data points (*P *= 0.03). Values are means ± SEs (*n* = 3).

In samples collected from lines under WD conditions, IL 4‐1 showed a lower TD than the other lines (Figure 2a). Leaves that developed under WD conditions showed a higher TD in ILs 4‐1 and 11‐3, compared with leaves developed under WW conditions. There were no significant differences for SD between lines under WD conditions (Figure S3). When TD and SD were measured in terms of area unit, we observed a consistently low TD in IL 4‐1 compared with ILs 10‐2 and 11‐3, and there were no differences for SD between lines or treatments (Figure S4). The *T*/*S* ratio was significantly lower in IL 4‐1 compared with IL 11‐3 (Figure 2b). Although differences in *T*/*S* between WW and WD plants were not significant, there was a trend for higher *T*/*S* under WD for IL 11‐3 (*P* < 0.1). In leaves developed under WD conditions, SS showed no significant differences between lines, but IL 4‐1 had lower SS in leaves developed under WD conditions compared with leaves developed under WW conditions (Figure 2c). For the rest of the lines, no significant differences were observed when comparing WW and WD conditions. We observed no relationship between TD and SD when all data points were considered together, but we observed an inverse association between TD and SD in WD plants (*R* ^2^ = 0.94; *P* = 0.03; Figure 2d).

### Comparative analysis of morphological parameters and water status under WW and WD conditions in the field

We evaluated the effects of differences in the densities of trichomes on water status of plants under WW and WD conditions in the field. We observed a lower midday leaf water potential (Ψ_leaf_) in IL 10‐2 compared with M82 and IL 11‐3 under WW conditions (Table 1). Under WD conditions, IL 11‐3 had higher Ψ_leaf_ than IL 10‐2. All lines showed a lower Ψ_leaf_ under WD conditions except for IL 11‐3, where no difference was observed.

**Table 1:** Morphological and water status characterisation of lines M82, 4‐1, 10‐2 and 11‐3 under well‐watered (WW) and water deficit (WD) conditions in the field. Midday leaf water potential (*Ψ*_leaf_), leaf mass per area (LMA), leaf thickness (LT) and plant‐level water use efficiency (WUE_b_) are shown. Values are means ± SE of three to four replicates per line and treatment. Different letters denote statistically significant differences by Tukey analysis (*P *<* *0.05) within each treatment between lines, and asteriks denote statistically significant differences (*P *<* *0.05) between treatments for each line

Acc.	WW				WD			
	Ψ_leaf_	LMA	LT	WUE_b_	Ψ_leaf_	LMA	LT	WUE_b_
	MPa	g m^−2^	mm	g kg^−1^ H_2_O	MPa	g m^−2^	mm	g kg^−1^ H_2_O
M‐82	−0.74 ± 0.04^a^	79.45 ± 9.56^a^	0.70 ± 0.05^ab^	0.76 ± 0.12^a^	−1.01 ± 0.07^ab^*	96.01 ± 3.41^a^	0.76 ± 0.04^ab^	1.14 ± 0.09^ab^*
4‐1	−0.86 ± 0.06^ab^	58.12 ± 4.33^a^	0.63 ± 0.03^b^	0.74 ± 0.04^a^	−1.18 ± 0.05^ab^*	63.83 ± 6.69^b^	0.66 ± 0.05^ab^	0.79 ± 0.02^b^
10‐2	−0.99 ± 0.04^b^	82.37 ± 6.58^a^	0.69 ± 0.01^ab^	0.89 ± 0.1^a^	−1.21 ± 0.07^b^*	98.65 ± 5.72^a^	0.81 ± 0.02^a^*	1.12 ± 0.17^ab^
11‐3	−0.74 ± 0.07^a^	71.20 ± 9.45^a^	0.80 ± 0.03^a^	0.85 ± 0.05^a^	−0.93 ± 0.08^a^	64.18 ± 6.35^b^	0.63 ± 0.03^b^*	1.36 ± 0.06^a^*

The leaf mass area (LMA) was lower in ILs 4‐1 and 11‐3 under WD conditions compared with the other lines, but no differences amongst lines under WW conditions or between WW and WD treatments were observed (Table 1). Leaf thickness (LT) was higher in IL 11‐3 compared with IL 4‐1 under WW conditions, but under WD conditions LT in IL 11‐3 was significantly lower than in IL 10‐2 (Table 1).

Whole‐plant water use efficiency (WUE_b_) showed no differences between lines under WW conditions (Table 1). Under WD conditions, WUE_b_ was lower in IL 4‐1 compared with IL 11‐3. In M82 and IL 11‐3, WUE_b_ was higher in WD plants compared with WW plants.

### Comparative analysis of photosynthetic parameters under WW and WD conditions in the field

The parameters *A*_N_, *C*_24h_, *g*_s_, *g*_m_, *g*_tot_, *V*_cmax_ and WUE_i_ showed no significant differences between lines under WW conditions in the field (Table 2). In contrast, under WD conditions, IL 4‐1 had a significantly lower WUE_i_ than the other lines. IL 11‐3 had a significantly higher WUE_i_ in WD compared with WW conditions (Table 2).

**Table 2:** Photosynthetic characterization of lines M82, 4‐1, 10‐2 and 11‐3 under well‐watered (WW) and water deficit (WD) conditions in the field. Net photosynthetic rate (*A*_N_), daily carbon fixation rate (*C*_24h_), stomatal conductance (*g*_*s*_), mesophyll conductance (*g*_*m*_), total leaf conductannce (*g*_tot_), maximum velocity of Rubisco carboxylation (*V*_cmax_) and intrinsic water use efficiency (WUE_i_) are shown. Values are means ± standard error of four replicates per line and treatment. Different letters denote statistically significant differences by Tukey analysis (*P *<* *0.05) within each treatment between lines, and asteriks denote statistically significant differences (*P *<* *0.05) between treatments for each line

Acc.	*A*_N_	*C*_24h_	*g*_s_	*g*_m_	*g*_tot_	*V*_cmax_	WUE_i_
	μmol CO_2_ m^−2^ sec^−1^	mol m^−2^ day^−1^	mol H_2_O m^−2^ sec^−1^	mol CO_2_ m^−2^ sec^−1^	mol CO2 m^−2^ sec^−1^	μmol CO2 m^−2^ sec^−1^	μmol CO_2_ mol^−1^ H_2_O
WW							
M‐82	13.22 ± 1.16^a^	0.34 ± 0.03^a^	0.222 ± 0.046^a^	0.087 ± 0.015^a^	0.049 ± 0.006^a^	304.05 ± 57.19^a^	66.22 ± 11.52^a^
4‐1	15.31 ± 1.35^a^	0.34 ± 0.08^a^	0.272 ± 0.027^a^	0.088 ± 0.004^a^	0.058 ± 0.003^a^	349.38 ± 107.21^a^	57.09 ± 4.68^a^
10‐2	16.87 ± 1.26^a^	0.42 ± 0.04^a^	0.229 ± 0.026^a^	0.123 ± 0.015^a^	0.063 ± 0.004^a^	335.96 ± 49.79^a^	75.88 ± 7.33^a^
11‐3	15.82 ± 1.43^a^	0.44 ± 0.05^a^	0.243 ± 0.026^a^	0.102 ± 0.017^a^	0.061 ± 0.010^a^	336.77 ± 38.46^a^	65.60 ± 2.11^a^
WD							
M‐82	11.52 ± 0.80^a^	0.31 ± 0.04^a^	0.165 ± 0.021^a^	0.084 ± 0.010^a^	0.046 ± 0.005^a^	271.97 ± 22.89^a^	71.44 ± 4.26^ab^
4‐1	12.53 ± 0.80^a^	0.30 ± 0.03^a^	0.233 ± 0.009^a^	0.083 ± 0.016^a^	0.052 ± 0.006^a^	217.25 ± 26.34^a^	53.74 ± 2.76^b^
10‐2	13.20 ± 1.59^a^	0.38 ± 0.03^a^	0.184 ± 0.014^a^	0.101 ± 0.018^a^	0.052 ± 0.006^a^	253.63 ± 49.58^a^	71.60 ± 5.29^ab^
11‐3	15.57 ± 2.38^a^	0.39 ± 0.03^a^	0.187 ± 0.034^a^	0.143 ± 0.044^a^	0.061 ± 0.013^a^	321.88 ± 52.84^a^	84.52 ± 6.92^a^*

The leaf carbon isotope composition (δ^13^C) showed no significant differences between lines within each treatment or between treatments for any line; however, leaf δ^13^C was correlated with WUE_i_ (*R* ^2^ = 0.67, *P* = 0.01; Figure S5a). We also observed a tight positive correlation (*R* ^2^ = 0.66, *P* = 0.01) between WUE_i_ and WUE_b_ (Figure S5b).

### Relationships between epidermal features and photosynthetic parameters

**Figure 3:**
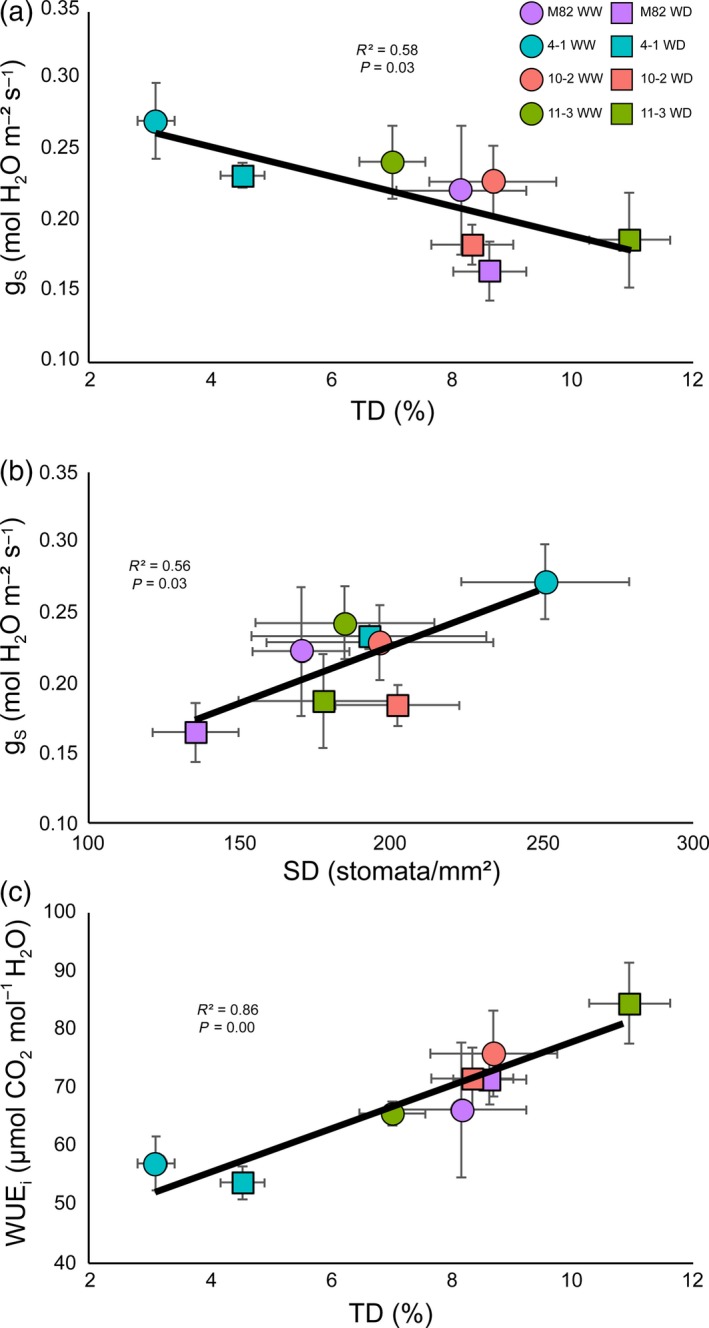
Relationships between trichome and stomatal densities and photosynthetic parameters in lines M82, 4‐1, 10‐2 and 11‐3: (a) inverse correlation between trichome density and stomatal conductance (*g*_s_); (b) positive association between stomatal density and stomatal conductance (*g*_s_); and (c) positive association between trichome density and intrinsic water use efficiency (WUE_i_). Correlation coefficients and *P* values calculated by Pearson's test are displayed in each graph. Purple markers represent M82, turquoise markers represent 4‐1, red markers represent 10‐2 and green markers represent 11‐3. Circles represent values under well‐watered (WW) conditions in the field and squares represent values under water‐deficit (WD) conditions in the field. Values are means ± SEs (*n* = 3–4).

**Figure 4:**
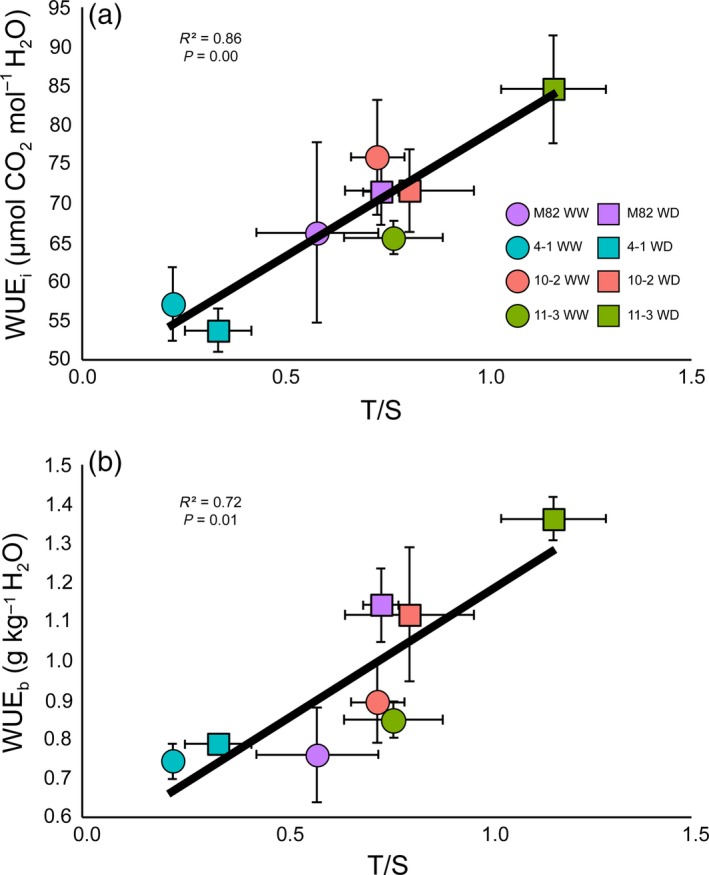
Relationship between trichome/stomata ratios and photosynthetic parameters in lines M82, 4‐1, 10‐2, and 11‐3: (a) correlation between trichome/stomata ratio (*T*/*S*) and intrinsic water use efficiency (WUE_i_); and (b) correlation between *T*/*S* and plant‐level water use efficiency (WUE_b_). Correlation coefficients, calculated by Pearson's test (*P *< 0.001 for a; *P *< 0.01 for b), are shown in each graph. Purple markers represent M82, turquoise markers represent 4‐1, red markers represent 10‐2 and green markers represent 11‐3. Circles represent values from well‐watered (WW) conditions in the field and squares represent values from water‐deficit (WD) conditions in the field. Values are means ± SEs (*n* = 3–4).

We found an inverse correlation between TD and *g*_s_ (*R* ^2^ = 0.58, *P* = 0.03; Figure 3a), and we also observed a positive correlation between SD per unit area and *g*_s_ (*R* ^2^ = 0.56, *P* = 0.03; Figure 3b). Therefore, changes in the density of trichomes and stomata had opposite effects at the gas‐exchange level. Importantly, TD was positively correlated with WUE_i_ (*R* ^2^ = 0.88, *P* = 0.00; Figure 3c). SD showed no correlation with WUE_i_. As expected from the observed relationship between WUE_i_ and WUE_b_ (Figure S5b), TD was positively correlated with WUE_b_ (*R* ^2^ = 0.59, *P* = 0.03; Figure S6a). Interestingly, SD was negatively correlated with WUE_b_ (*R* ^2^ = 0.50, *P* < 0.05; Figure S6b). The correlation between TD and WUE_i_ and WUE_b_ was maintained when TD was expressed per unit area (Figure S7), but this was not the case for the correlation between SD and WUE_b_, which was only observed when SD was expressed as a percentage of epidermal cells. Finally, a strong positive association was found between *T*/*S* and WUE_i_ (*R* ^2^ = 0.86, *P* = 0.00; Figure 4a) and WUE_b_ (*R* ^2^ = 0.72, *P* = 0.01; Figure 4b).

## Discussion

### The development of leaf trichomes is influenced by water availability

We observed a 10‐ to 15‐fold higher TD in leaves grown under field conditions compared with glasshouse‐grown plants (Figures 1a and S1). These differences were likely to have resulted from differences in the age of the plant as well as changes in environmental conditions between the glasshouse and the field. Trichome development is reported to change with the age of the plant (Telfer *et al*., 1997; Vendemiatti *et al*., 2017), with higher TD observed in the late leaves of tomato (Gurr and McGrath, 2001). Moreover, environmental factors, such as temperature, photoperiod, light intensity or soil humidity have direct effects on trichome development in several species, including tomato (Wellso and Hoxie, 1982; Gianfagna *et al*., 1992; Chien and Sussex, 1996; Souza *et al*., 2016). Despite the dramatic change between environmental conditions in the glasshouse and the field, the relative differences in TD were conserved between lines (Figure 1a), indicating strong genetic control of trichome development in the selected ILs. We observed a 1.5‐ to 5.0‐fold higher SD in field‐grown plants (Figure 1b). These differences could also be a function of the age of the plant when leaves were sampled (Ceulemans *et al*., 1995) as well as the environmental conditions (Beerling and Chaloner, 1993; Rogiers *et al*., 2011). Unlike the observation for TD, relative differences in SD between lines were not conserved in different environments, pointing to the differential regulation of TD and SD.

We assessed the changes in leaf anatomy in WD plants compared with WW plants. We observed a higher TD in ILs 4‐1 and 11‐3 under WD conditions compared with WW conditions (Figure 2a). Increases in TD with herbivore and water stress have been reported previously in several species (Traw and Bergelson, 2003; Bjorkman *et al*., 2008; Fu *et al*., 2013), as part of the adaptive stress response. In fact, transcriptomic studies in water‐stressed *Arabidopsis thaliana* plants showed an upregulation of genes related to trichome initiation and morphogenesis (TT8, BRICK1, KAK), but not of genes involved in stomatal initiation (Bechtold *et al*., 2016). Not all the lines in this study showed uniform responses, with IL 4‐1 showing bigger changes upon WD treatment (lower SS, higher TD) (Figure 2). This could be explained by a greater inability of IL 4‐1 to control water loss, as indicated by its lower WUE_b_ and WUE_i_ (Tables 1 and 2), leading to more severe physiological stress in this line and, subsequently, a stronger response to WD. In any case, these leaf adaptive changes did not account for an increase in WUE (Tables 1 and 2), probably because of a lower overall TD in IL 4‐1.

We observed an inverse association between trichome density (TD) and stomatal density (SD) only under WD conditions (Figure 2d), in agreement with previous reports in *Nicotiana tabacum* (tobacco) and tomato (Glover *et al*., 1998; Glover, 2000). Developmentally, trichomes and stomata originate from protodermal cells (Morohashi and Grotewold, 2009; Pillitteri and Dong, 2013), and the inverse association observed suggests that the regulation of their development might be linked. Similar relationships have been found in trichome mutants of *A. thaliana* (Bean *et al*., 2002), where altered trichome phenotypes affected stomatal patterning. In aubergine, increases in TD have been associated with increases in SD (Fu *et al*., 2013), in contrast to our observations, indicating that there may be different developmental associations even between related *Solanum* species. The correlation was not observed when TD and SD per area were used, suggesting that TD and SD as percentage of epidermal cells might give a better representation of the developmental changes in the epidermis. The fact that TD and SD were not correlated when both WW and WD plants were considered (Figure 2d) might be a result of the lack of genetic differences in SD, as only TD was clearly different between the assessed lines (Figures 1b and S1b). However, the observed TD–SD association suggests that the developmental response of the leaf to drought stress involves changes in the determination of cell fate in the whole epidermal tissue, simultaneously affecting TD and SD, and this might occur through different regulatory mechanisms under different water regimes. In conclusion, we observed an important effect of water availability on leaf anatomy and the determination of epidermal features.

### Variation amongst the ILs and the potential for developing drought‐tolerant varieties

The highly inbred tomato cultivar M82 has traditionally been used as a check variety in breeding programmes (Grandillo *et al*., 1999) and as a reference cultivar for scientific research, used in relation to the response to water stress as a drought‐sensitive cultivar (Iovieno *et al*., 2016), in contrast with the drought‐tolerant *S. pennellii* (Egea *et al*., 2018). The IL population has been used extensively for functional and physiological studies (Steinhauser *et al*., 2011; Chitwood *et al*., 2013; de Oliveira Silva *et al*., 2018), and the natural variation within the IL population provides an excellent platform to investigate the role of differences in epidermal features on performance under water stress.

The intrinsic water use efficiency (WUE_i_) is an important target for crop improvement with respect to drought tolerance, although it needs to be considered carefully as it might not be directly related to improved fruit productivity (Blum, 2005, 2009). We observed a lower WUE_i_ for IL 4‐1 under WD conditions compared with the other lines under study (Table 2), whereas WUE_i_ in IL 11‐3 was higher under WD compared with WW conditions (Table 2). This increase in WUE_i_ has been reported in drought‐tolerant varieties in several crops (Guha *et al*., 2010; Fracasso *et al*., 2016; Liu *et al*., 2016), although it might not always have a positive effect on fruit yield. Interestingly, none of the ILs under study were considered before for WUE improvement as they showed no differences in δ^13^C compared with M82 (Xu *et al*., 2008), in agreement with our results, so this epidermis‐based analysis provides an alternative path for increased WUE. Therefore, the genomic region introgressed from *S. pennellii* in IL 11‐3 could be selected to generate more water use efficient tomato cultivars.

The accuracy of WUE_i_ as a measure of drought tolerance has been questioned because of the lack of correlation with whole‐plant measurements of WUE (Medrano *et al*., 2015). In fact, in newer tomato cultivars, a decrease in WUE_i_, driven by selection under high light levels and well‐watered conditions, has been reported to be accompanied by increases in agronomic WUE (yield per water used; Barrios‐Masias and Jackson, 2014). Therefore, biomass‐based parameters, specifically plant‐level water use efficiency (WUE_b_), were also investigated in this study, although specific differences in fruit production or harvest index between ILs were not considered (Caruso *et al*., 2016). WUE_b_ under WD was lower in IL 4‐1 than in IL 11‐3 (Table 1). Moreover, M82 and IL 11‐3 plants grown under WD conditions had higher WUE_b_ values compared with WW plants (Table 1). The observed WUE_i_–WUE_b_ correlation (Figure S5b) supports the use of WUE_i_ as a measure of drought tolerance under our experimental conditions. In a similar way, leaf δ^13^C has been used as a marker of the WUE of a plant (Farquhar and Richards, 1984), and in tomato and *S. pennellii* it has been reported to correlate with WUE_b_ (Martin and Thorstenson, 1988). In this work, we found a correlation between leaf δ^13^C and WUE_i_ (Figure S5a), suggesting that leaf δ^13^C can be used as a tool for selection of high‐WUE_i_ lines in tomato. We did not observe a significant correlation between leaf δ^13^C and WUE_b_, however, which might hinder the applicability of our findings.

### The role of epidermal features during WD

Water‐stress responses involve changes in leaf anatomy, including changes in the leaf thickness affecting CO_2_ diffusion (Niinemets *et al*., 2009; Galmes *et al*., 2013) and epidermal features, where there has been a focus on the stomata because of their direct role in gas exchange and transpiration (Galmés *et al*., 2007a; Xu and Zhou, 2008). Although we observed differences in LT in ILs 4‐1 and 11‐3 when WW and WD plants were compared (Table 1), no correlation was found between LT and any gas exchange parameter. Importantly, we observed a significant, inverse correlation between *g*_s_ and SD per area (Figure 3), but not between *g*_s_ and SD as a percentage of epidermal cells. This result suggests that SD per area should be used preferentially when assessing gas exchange parameters.

Our findings also support a major role for trichomes in gas exchange and the determination of WUE, however. The correlation observed between TD and WUE_i_ and WUE_b_ (Figures 3b, S6a and S7) suggests that TD has a positive effect in terms of drought tolerance. The lack of differences in trichome types or trichome length in the lines under study (Figure S2) indicates that the observed effects are linked to TD rather than to other trichome parameters. Several roles have been suggested for trichomes in plant drought tolerance (Galmés *et al*., 2007a; Boughalleb and Hajlaoui, 2011). On the basis that TD is negatively associated with *g*_s_ (Figure 3a), our data suggest that trichomes in tomato might play a role in avoiding excessive water loss by changing the resistance of the boundary layer, as proposed in previous studies (Guerfel *et al*., 2009; Mo *et al*., 2016). Another possible explanation for the correlation between TD and WUE involves the mutually exclusive developmental association between trichomes and stomata. Increased trichome initiation as part of the response to drought (a possibility supported by expression analysis in Arabidopsis; Bechtold *et al*., 2016), could lead to lower SD that could lead to an improved WUE. Genes involved in ABA signalling, known to play a role in the drought response, are expressed in trichomes (Ren *et al*., 2010; Daszkowska‐Golec, 2016). For example, the tomato homologue of the WRKY transcription factor ABA OVERSENSITIVE 3 (AtABO3) (Ren *et al*., 2010) or the bZIP transcription factor ABRE BINDING FACTOR 1 (AtABF1) (Yoshida *et al*., 2015) are both expressed in trichomes in tomato according to the available RNAseq data (Spyropoulou *et al*., 2014). Changes in TD could lead to changes in the transcript abundance of these or other ABA‐related factors. In fact, the expression level of SlABO3 is slightly higher in leaves of IL 11‐3 compared with leaves of ILs 4‐1 and 10‐2 according to the available RNAseq data (Chitwood *et al*., 2013), and SlABF1 is located in the genomic region introgressed from *S. pennellii* in IL 11‐3, indicating a possible role for trichome‐expressed ABA‐related genes in the observed drought response.

Whole‐plant water use efficiency (WUE_b_) was also correlated with SD (Figure S6b), although the impact of SD on WUE was lower than that of TD, because there was no correlation between WUE_i_ and SD at the leaf level, and when correlation coefficients for TD–WUE_b_ and SD–WUE_b_ were compared, the effect of SD on WUE_b_ was lower than that of TD (Figures 3 and S6). The fact that both stomata and trichomes were involved in the drought response was not surprising, given the developmental link that we observed under WD conditions (Figure 2d) and the direct role of stomata in gas exchange (Figure 3b). In fact, the ratio of trichomes to stomata (*T*/*S*), which gives information about the relationship between both structures, showed a strong correlation with both WUE_i_ and WUE_b_ (Figure 4). In addition to this, *T*/*S* is unitless and is not expressed either in terms of leaf area or percentage of epidermal cells, thereby overcoming the differences observed in correlations between developmental and photosynthetic traits. It is interesting to note that *T*/*S* becomes a more prominent parameter under WD conditions, when both TD and SD change together (Figure 2d), whereas under WW conditions, when there is no correlation between them (Figure 2d) or significant changes between lines (Figure S4), the genotype‐specific TD is likely to be the main player in the relation between epidermis and WUE. In conclusion, *T*/*S* plays an important role in the efficiency by which water is used by tomato, and differences in *T*/*S* could be used to develop more drought‐tolerant tomato varieties.

## Experimental procedures

### Preliminary characterization of epidermal cells

From a visual inspection of 76 *S. lycopersicum* cv. M82 × *S. pennellii* ac. LA716 ILs (Eshed and Zamir, 1995), grown under glasshouse conditions, we selected four lines (ILs 4‐1, 10‐2 and 11‐3, and M82) displaying a clear visual trichome phenotype. IL 4‐1 (LA4048) had an apparent lower trichome density (TD) than the parental line M82, IL 10‐2 (LA4089) had a hairless‐like phenotype, and IL 11‐3 (LA4092) had an apparently higher TD compared with the parental line M82 (LA3475). We characterised the trichome densities of the adaxial and abaxial sides of leaves from the four lines under study. This analysis indicated similar values for TD on both sides of the leaf in all four lines and a strong correlation (*R* ^2^ = 0.92, *P* = 0.04) between the values on both sides of the leaf (Figure S8), which allowed us to work with values on the adaxial surface in all future investigations.

The epidermal features of these four lines were characterised further using scanning electron microscopy (SEM) of plants grown both under glasshouse and field conditions. For glasshouse assays, three or four plants per line were grown under water field capacity at the John Innes Centre (https://www.jic.ac.uk), using natural light with an average temperature of between 20 and 22°C. The terminal leaflet of the first leaf of 4‐week‐old plants was excised, and inter‐vein sections of approximately 0.5 × 0.5 cm were taken as samples. These sections were vacuum‐fixed in a glutaraldehyde 2.5% cacodylate solution and dehydrated through an ethanol series. Samples were dried in a Leica CPD300 critical‐point dryer (Leica Microsystems, http://www.leica.com), to avoid the collapse of trichomes, and were gold‐coated before imaging in a Zeiss Supra 55 VP SEM (Zeiss, https://www.zeiss.com) at 20°C and under high‐vacuum conditions, generating between eight and 15 micrographs of approximately 0.3 mm^2^ per sample of the adaxial surface.

The epidermal features of these four lines were characterised further using scanning electron microscopy (SEM) of plants grown both under glasshouse and field conditions. For glasshouse assays, three or four plants per line were grown under water field capacity at the John Innes Centre (https://www.jic.ac.uk), using natural light with an average temperature of between 20 and 22°C. The terminal leaflet of the first leaf of 4‐week‐old plants was excised, and inter‐vein sections of approximately 0.5 × 0.5 cm were taken as samples. These sections were vacuum‐fixed in a glutaraldehyde 2.5% cacodylate solution and dehydrated through an ethanol series. Samples were dried in a Leica CPD300 critical‐point dryer (Leica Microsystems, http://www.leica.com), to avoid the collapse of trichomes, and were gold‐coated before imaging in a Zeiss Supra 55 VP SEM (Zeiss, https://www.zeiss.com) at 20°C and under high‐vacuum conditions, generating between eight and 15 micrographs of approximately 0.3 mm^2^ per sample of the adaxial surface.

Characterisation under field conditions was carried out in the experimental plot at the University of the Balearic Islands (UIB, http://www.uib.eu). The environmental conditions during plant growth are detailed in the next section. Six plants were sampled for each line under study. Terminal leaflets of fully expanded leaves at the same position were excised and sections of 0.5 × 0.5 cm were taken as samples. The adaxial surface of these sections was imaged at 20°C, without coating, inside a Hitachi 3400N variable pressure SEM (Hitachi High‐Tecnhology, https://www.hitachi-hightech.com). We generated between eight and 10 micrographs of approximately 0.3 mm^2^ per sample, and trichomes, stomata and pavement cells were counted manually. In both analyses, trichome and stomatal densities were expressed both as a percentage of epidermal cells and as density per area. Trichomes were classified in types and had their length measured. For trichome density, all types of trichome were considered together. The ratio of trichomes to stomata was calculated as trichome density divided by stomatal density.

### Field growth conditions and drought treatment

Seeds from the four lines were germinated and grown for 4 weeks in glasshouses at the University of the Balearic Islands (UIB) with natural light and average maximum temperatures of 25°C in March–April 2016. Twelve plants per line were placed outdoors in the UIB experimental field for acclimation for 2 weeks before being transferred to 50‐L pots containing a mixture of bog peat‐based horticultural substrate (Prohumin‐Potting Soil Klasmann‐Deilmann; Projar, https://www.projar.es) and perlite (granulometry A13; Projar) in a 4 : 1 proportion (v/v). The environmental conditions from June to September 2016 were those typical for a Mediterranean summer: with average daily temperatures of 22.8 ± 0.3, 25.7 ± 0.3, 25.0 ± 0.2 and 22.7 ± 0.4°C; average daily minimum temperatures of 16.5 ± 0.5, 20.3 ± 0.3, 19.5 ± 0.4 and 17.8 ± 0.4°C; and average daily maximum temperatures of 31.1 ± 0.4, 35.5 ± 0.4, 33.5 ± 0.3 and 34.7 ± 0.6°C, respectively, for June, July, August and September. Plants were watered to field capacity every other day and fertilised weekly with 50% Hoagland's solution for 2 months before beginning the drought treatment.

From 11 July, plants were subjected to two different water regimes: WW and WD. Watering regimes and plant water consumption were monitored by weighing and watering the potted plants every 2 days. For the WW treatment, four plants per line were maintained at field capacity, with a pot water content ranging between 100% field capacity just after irrigation (corresponding to 9.3 L of water per pot) and 69.3 ± 0.1% field capacity (on average throughout the treatment period) (Figure S9). For the WD treatment, the irrigation of four plants was progressively reduced until halving the pot water content compared with the WW plants. Then, WD plants were maintained at a pot water content ranging between 30.3 ± 0.1% field capacity before irrigation and 46.3 ± 0.1% field capacity after irrigation (Figure S9). The four remaining plants per line were used for biomass‐related measurements. The total water supplied and dry biomass of each plant was recorded upon experiment completion (Table S2). Three weeks were allowed from treatment application for the development of new leaves under the new water regime before any measurement was performed.

All leaf‐based measurements and samples were taken from the terminal leaflets of the youngest fully expanded leaves generated after application of the treatment. Epidermal features (trichome and stomatal densities, stomatal size and ratio of trichomes to stomata) were evaluated as described for glasshouse‐grown plants in the ‘Preliminary characterization of plant material’ section.

### Plant water status and growth‐related measurements

Plant water status was measured as midday leaf water potential (Ψ_leaf_) (*n* = 4 per line and treatment) using a Scholander pressure chamber (Soilmoisture Equipment Corp., https://www.soilmoisture.com), as described by Turner (1988).

For the calculation of WUE_b_, four plants per line were harvested at the time of drought treatment. Seventy‐four days after treatment application, four plants per line and per treatment (WW and WD) were harvested. Leaves, shoots and roots were oven‐dried separately at 60°C and weighed (dry weight, DW). Biomass production during the drought treatment was calculated as the difference between the DW of the plants harvested at the end of the experiment (DW_final_) and the *DW* of the plants harvested before the treatment (DW_initial_). Water consumption was monitored every other day by weighing the pots containing the plants, and the total water consumption (TWC) of each plant was estimated from these values. WUE_b_ was calculated as follows: WUE_b_ = (DW_final_ − DW_initial_)/TWC.

### Leaf morphological determinations

Leaf thickness (LT) was determined for the middle part of the terminal leaflet of a young fully‐expanded leaf with callipers, avoiding regions of the leaf with major veins. Leaf mass area (LMA) was calculated from the same terminal leaflets, as the dry mass to leaf area ratio. Dry mass was measured by weighing after oven‐drying leaves at 60°C for 48 h. The leaf area was digitally measured from pictures of the leaves using imagej 1.49 (National Institutes of Health, https://imagej.nih.gov/ij/) before drying. Both LT and LMA were measured for one leaf per plant (*n* = 4 per line and treatment).

### Leaf gas exchange and chlorophyll *a* fluorescence

Measurements were performed with an open infrared gas‐exchange analyser equipped with a leaf chamber fluorometer (Li‐6400‐40; LI‐COR, https://www.licor.com) from 09:00 to 12:00 h and from 16:00 to 19:00 h in the first 2 weeks of August 2016. Preliminary tests confirmed non‐significant differences between morning and afternoon measurements. The conditions in the chamber consisted of leaf temperatures of 31–33°C, a vapour pressure deficit of 2.0–3.0 kPa and an air flow of 500 μmol (air) min^−1^. For net CO_2_ assimilation rate–substomatal CO_2_ concentration (*A*_N_–*C*_i_) curves, the ambient concentration of CO_2_ in the chamber (*C*_a_) was set at 400, 0, 50, 100, 200, 300, 600, 900, 1500, 2000 and 400 μmol CO_2_ mol^−1^ air, at a saturating photosynthetic photon flux density (PPFD) of 1500 μmol m^−2^ sec^−1^ (with 10% blue light), allowing 4 min between measurements for the chamber to reach a steady state. Corrections for CO_2_ leakage in and out of the leaf chamber of the Li‐6400‐40 were applied to all gas‐exchange data, as described by Flexas *et al*. (2007).

Mesophyll conductance to CO_2_ (*g*_m_) was estimated according to (Harley *et al*., 1992) as:

$$g_{m}=A_{N}/C_{i}-\left(\Gamma^{*}\left[\mathrm{ETR}+8\left(A_{N}+R_{L}\right)\right]/\left[\mathrm{ETR}-4\left(A_{N}+R_{L}\right)\right]\right),$$

where Γ* is the chloroplast CO_2_ compensation point in the absence of day respiration, ETR is the electron transport rate and *R*_L_ is the rate of non‐photorespiratory CO_2_ evolution under light. ETR and *R*_L_ were calculated as described by Galmes *et al*. (2011). Γ* was retrieved from *in vitro*‐based measurements for *S. lycopersicum* by Hermida‐Carrera *et al*. (2016), but adjusted for the leaf temperature during the measurement.

Total leaf conductance (*g*_tot_) was calculated assuming the stomatal conductance (*g*_s_) and mesophyll conductance (*g*_m_) were in series, such that: *g*_tot _= 1/(1/*g*_s_ **+ 1/*g*_m_).

*A*_N_–*C*_i_ curves were transformed into *A*_N_‐chloroplastic CO_2_ concentration (*C*_c_) curves using estimated values of *g*_m_. From *A*_N_–*C*_C_ curves, the maximum velocity of Rubisco carboxylation (*V*_cmax_) was calculated as described by (Bernacchi *et al*., 2002), but using specific values of Rubisco kinetics for *S. lycopersicum* adjusted to the leaf temperature during the measurement (Hermida‐Carrera *et al*., 2016). The intrinsic water use efficiency (WUE_i_) was calculated as the ratio between the net photosynthetic rate (*A*_N_) and the stomatal conductance (*g*_s_), *A*_N_/*g*_s._

We determined the daily carbon fixation rate (*C*_24h_) by measuring the net CO_2_ exchange rate at 2‐h intervals over 24 h. These measurements were performed after drought treatment (*n* = 4 per line and per treatment) using an open infrared gas‐exchange analyser equipped with a clear chamber (Li‐6400‐40; LI‐COR). Three measurements were performed under ambient CO_2_ and light levels, averaged per plant and per time point. The daily fixation rate was calculated as the integral value for the curve generated by the point measurements.

### Leaf δ^13^C isotope composition

The dried leaves used to calculate *LMA* were ground to fine dust for the determination of carbon isotopic composition. Samples were subjected to combustion in an elemental analyser (Thermo Flash EA 1112 Series; ThermoFisher Scientific, http://www.thermofisher.com) and CO_2_ was injected into a continuous‐flow isotope ratio mass spectrometer (Thermo‐Finnigan Delta XP; ThermoFisher Scientific). Peach leaf standards (NIST 1547) were run every six samples. The standard deviation of the analysis was <0.2%.

### Statistical analysis

The differences between lines, treatments and interactions were assessed by univariate analysis of variance (anova). Significant differences between means were determined by a post‐hoc Tukey's test (*P* < 0.05). The relationship between variables in each experiment was determined by correlation coefficient (*R* ^2^). The analyses were performed using r 3.2.2 (R Core Team, https://www.r-project.org).

## Conflict of interest

The authors declare no conflicts of interest.

## Supporting information


**Figure S1**. Initial morphological characterization of lines M82, 4‐1, 10‐2 and 11‐3 grown under glasshouse (GH) and field conditions (F) before the onset of drought treatment.
**Figure S2**. Percentage of trichome types and trichome length in plants grown under glasshouse conditions.
**Figure S3**. Stomatal density in lines M82, 4‐1, 10‐2 and 11‐3 under water‐deficit (WD) and well‐watered (WW) conditions in the field.
**Figure S4**. Trichome and stomatal densities in lines M82, 4‐1, 10‐2 and 11‐3 under water‐deficit (WD) and well‐watered (WW) conditions in the field, expressed in terms of area.
**Figure S5**. Correlations between carbon isotope composition and intrinsic water use efficiency, and between intrinsic water use efficiency and plant‐level water use efficiency, in lines M82, 4‐1, 10‐2 and 11‐3.
**Figure S6**. Relationship between epidermal features and plant‐level water use efficiency (WUE_b_) in plants under well‐watered (WW) and water‐deficit (WD) conditions in the field.
**Figure S7**. Correlations between trichome density expressed per unit area and water use in lines M82, 4‐1, 10‐2 and 11‐3 under WW and WD conditions.
**Figure S8**. Trichome densities on abaxial and adaxial sides of leaves of lines M82, 4‐1, 10‐2 and 11‐3 grown under glasshouse conditions.
**Figure S9**. Evolution of the pot water content during the experiment for the well‐watered (WW, blue) and water‐deficit (WD, red) plants.

**Table S1**. Leaf morphological traits and photosynthetic characterization of the lines M82, 4‐1, 10‐2 and 11‐3 under field conditions before the onset of the drought treatment.
**Table S2**. Dry biomass and total water supplied to plants upon completion of the experiment for lines M82, 4‐1, 10‐2 and 11‐3.
