# Supplementary material for: The ratio of trichomes to stomata is associated with water use efficiency in Solanum lycopersicum (tomato)
Source: Plant J. 2018 Sep 5;96(3):607–19. doi: 10.1111/tpj.14055 (PMC6321981; doi:10.1111/tpj.14055)
Supplement: Supplementary file 1 — Figure S1. Initial morphological characterization of lines M82, 4‐1, 10‐2 and 11‐3 grown under glasshouse (GH) and field conditions (F) before the onset of drought treatment. Figure S2. Percentage of trichome types and trichome length in plants grown under glasshouse conditions. Figure S3. Stomatal density in lines M82, 4‐1, 10‐2 and 11‐3 under water‐deficit (WD) and well‐watered (WW) conditions in the field. Figure S4. Trichome and stomatal densities in lines M82, 4‐1, 10‐2 and 11‐3 under water‐deficit (WD) and well‐watered (WW) conditions in the field, expressed in terms of area. Figure S5. Correlations between carbon isotope composition and intrinsic water use efficiency, and between intrinsic water use efficiency and plant‐level water use efficiency, in lines M82, 4‐1, 10‐2 and 11‐3. Figure S6. Relationship between epidermal features and plant‐level water use efficiency (WUEb) in plants under well‐watered (WW) and water‐deficit (WD) conditions in the field. Figure S7. Correlations between trichome density expressed per unit area and water use in lines M82, 4‐1, 10‐2 and 11‐3 under WW and WD conditions. Figure S8. Trichome densities on abaxial and adaxial sides of leaves of lines M82, 4‐1, 10‐2 and 11‐3 grown under glasshouse conditions. Figure S9. Evolution of the pot water content during the experiment for the well‐watered (WW, blue) and water‐deficit (WD, red) plants. [file TPJ-96-607-s001.docx]

**
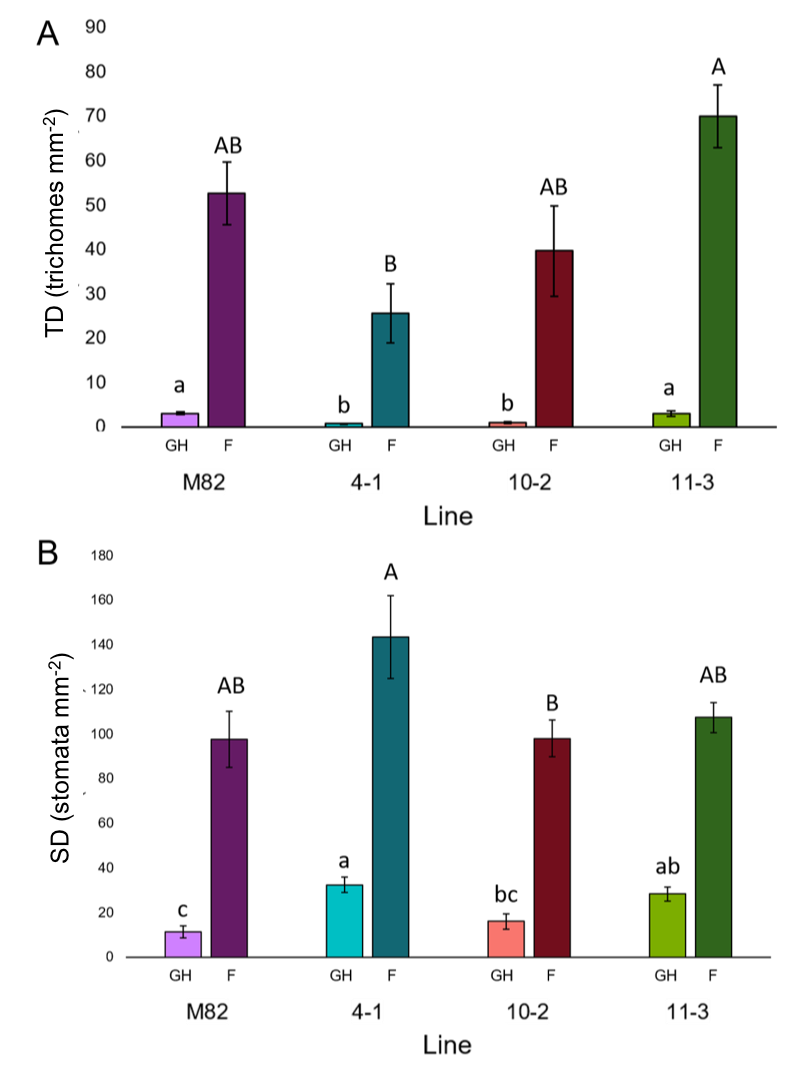
**

**Figure S1. Initial morphological characterisation of lines M82, 4-1, 10-2 and 11-3 grown under greenhouse (GH) and field conditions (F) before the onset of drought treatment.** (A) Trichome density (TD) and (B) stomatal density (SD) are expressed as mean ± SE of three to six replicates per line and treatment. TD and SD were calculated as number of trichomes or stomata per mm^2^. Different letters denote statistically significant differences by Tukey analysis (*P*<0.05) within greenhouse-grown plants (lower case) and field-grown plants (upper case). Purple bars represent M82, turquoise represents IL4-1, red represents IL10-2 and green IL11-3, with light and dark colours representing greenhouse (labelled GH) and field (labelled F) conditions, respectively.


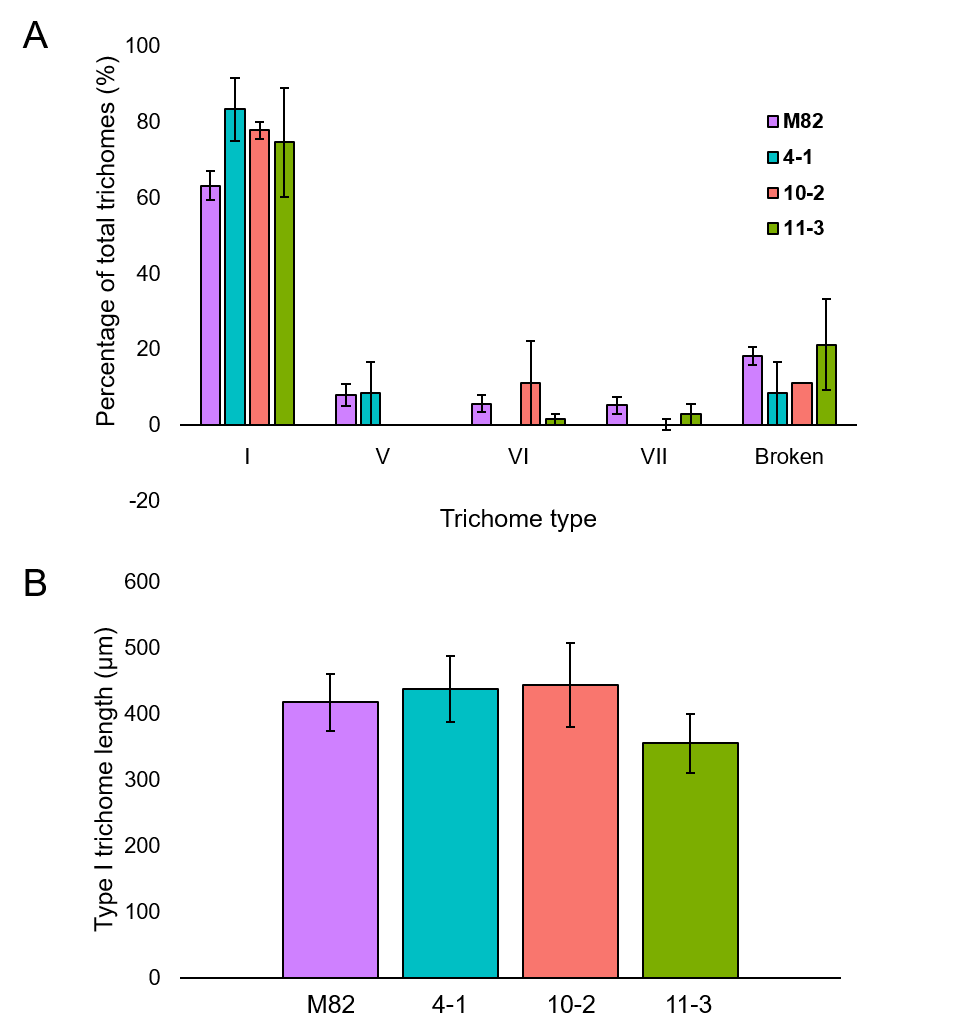


**Figure S2. Percentage of trichome types and trichome length in plants grown under greenhouse conditions.** A) Percentage of each type of trichome: type I-IV, type V, type VI, type VII and broken trichomes classified according to McDowell et al., 2011. B) Trichome length of type I trichomes, the most abundant type. No significant differences were identified by Tukey analysis (*P*<0.05) in any case. Purple bars represent M82, turquoise represents IL4-1, red represents IL10-2 and green represents IL11-3. Values are mean ± SE (n=3).


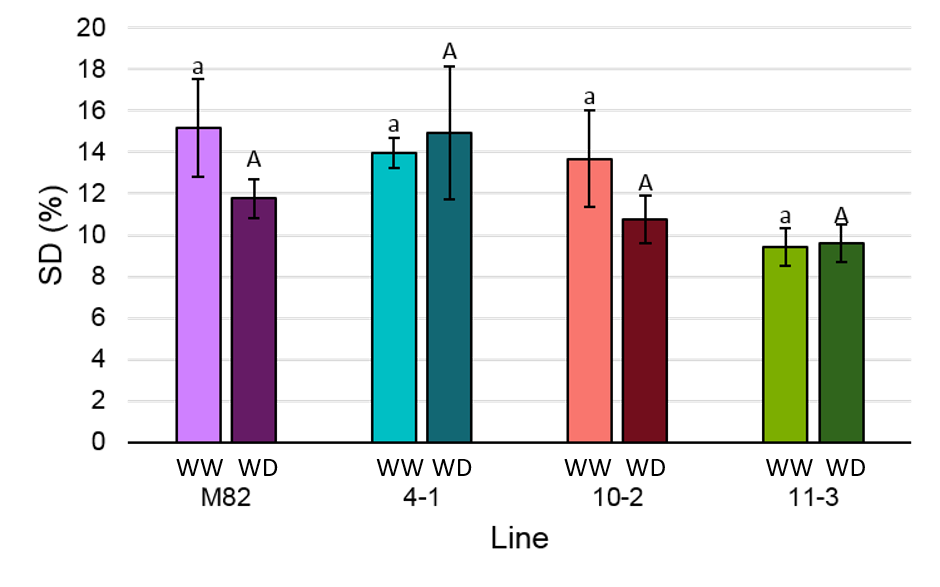


**Figure S3. Stomatal density in lines M82, 4-1, 10-2 and 11-3 under water deficit (WD) and well-watered (WW) conditions in the field.** No significant differences were identified by Tukey analysis (*P*<0.05) within WW plants (lower case) and WD plants (upper case), or between treatments for each line. Purple bars represent M82, turquoise represents IL4-1, red represents IL10-2 and green represents IL11-3, with light and dark colours representing WW and WD treatments, respectively. Values are mean ± SE (n=3).


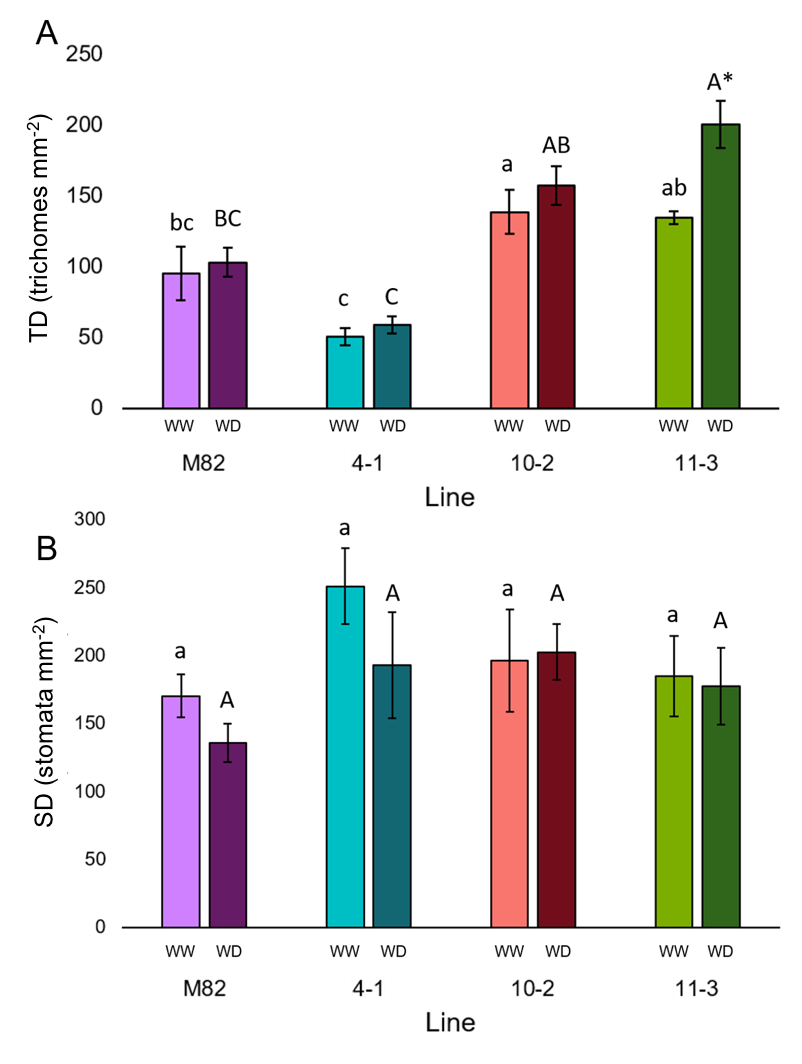


**Figure S4. Trichome and stomatal densities in lines M82, 4-1, 10-2 and 11-3 under water deficit (WD) and well-watered (WW) conditions in the field expressed in terms of area.** Different letters indicate significant differences (*P*<0.05) within WW plants (lower case) and WD plants (upper case). Stars indicate significant differences between treatments for each line (*P*<0.05). Significant differences were identified by Tukey analysis in each case. Purple bars represent M82, turquoise represents IL 4-1, red represents IL 10-2 and green represents IL 11-3, with light and dark colours representing WW and WD treatments, respectively. Values are mean ± SE (n=3).


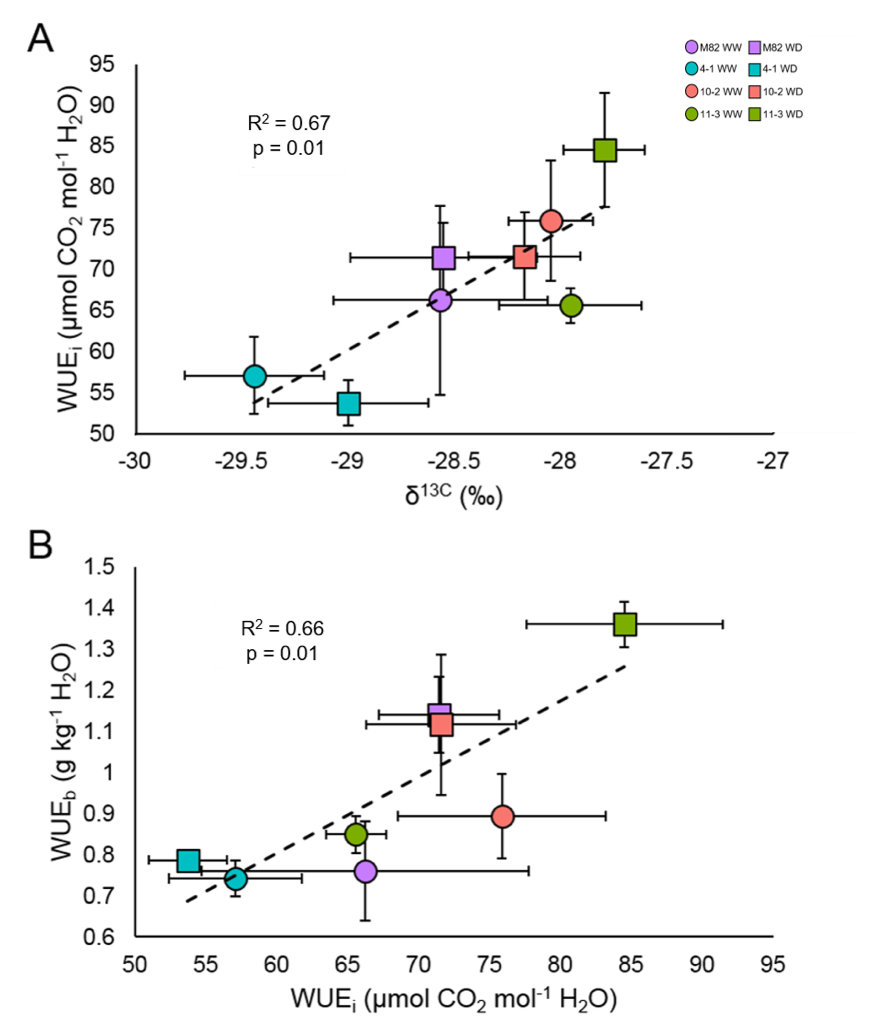


**Figure S5. Correlations between carbon isotope composition and intrinsic water use efficiency and between intrinsic water use efficiency and plant-level water use efficiency in lines M82, 4-1, 10-2 and 11-3.** (A) The correlation between leaf *δ^13C^* and intrinsic water use efficiency (WUE_i_) (B) The correlation between the intrinsic WUE_i_ and the plant-level water use efficiency (WUE_b_). Correlation coefficients and p-values by Pearson tests are shown in each graph. Purple markers represent M82, turquoise markers represent IL 4-1, red markers represent IL 10-2 and green markers represent IL 11-3. Circles represent WW values and squares represent WD values of field-grown plants. Values are means ± SE (n=4).


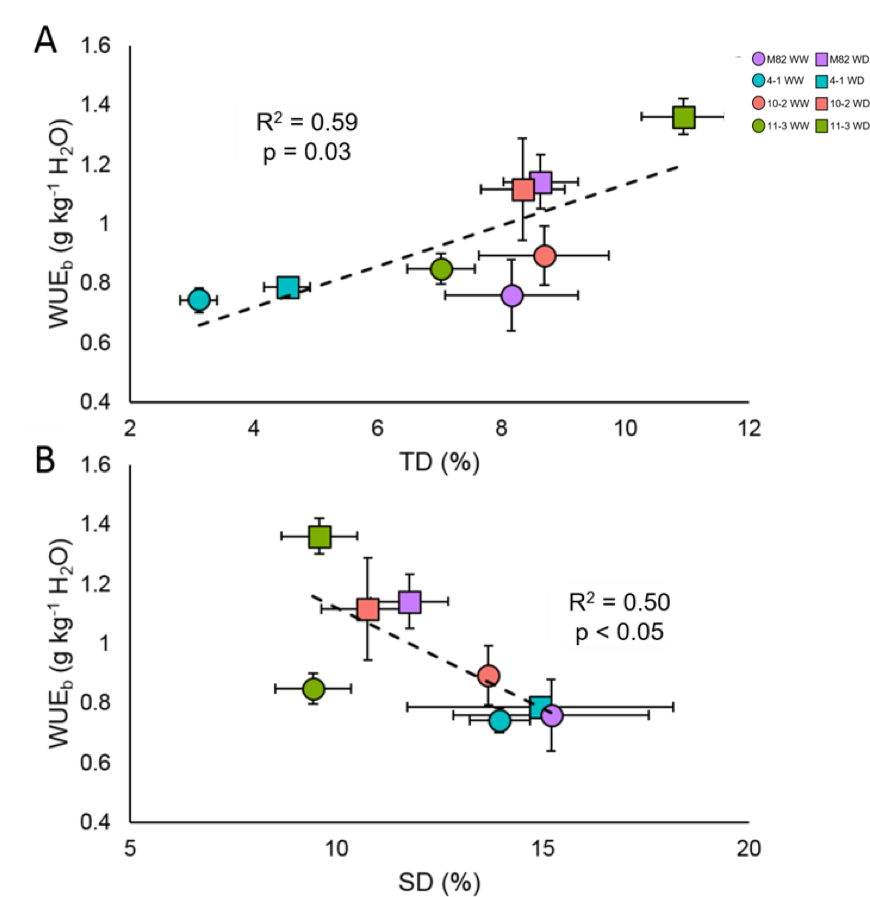


**Figure S6.** **Relationship between epidermal features and plant-level water use efficiency (WUE_b_) in plants under well-watered (WW) and water deficit (WD) conditions in the field.** (A) The correlation between trichome density and plant-level WUE_b_. (B) The correlation between stomatal density and plant-level WUE_b_. Correlation coefficients and p-values from Pearson tests are displayed in each graph. Purple markers represent M82, turquoise markers represent IL 4-1, red markers represent IL 10-2 and green markers represent IL 11-3. Circles represent values from WW conditions and squares represent values from WD conditions. Values are mean ± SE (n=3-4).


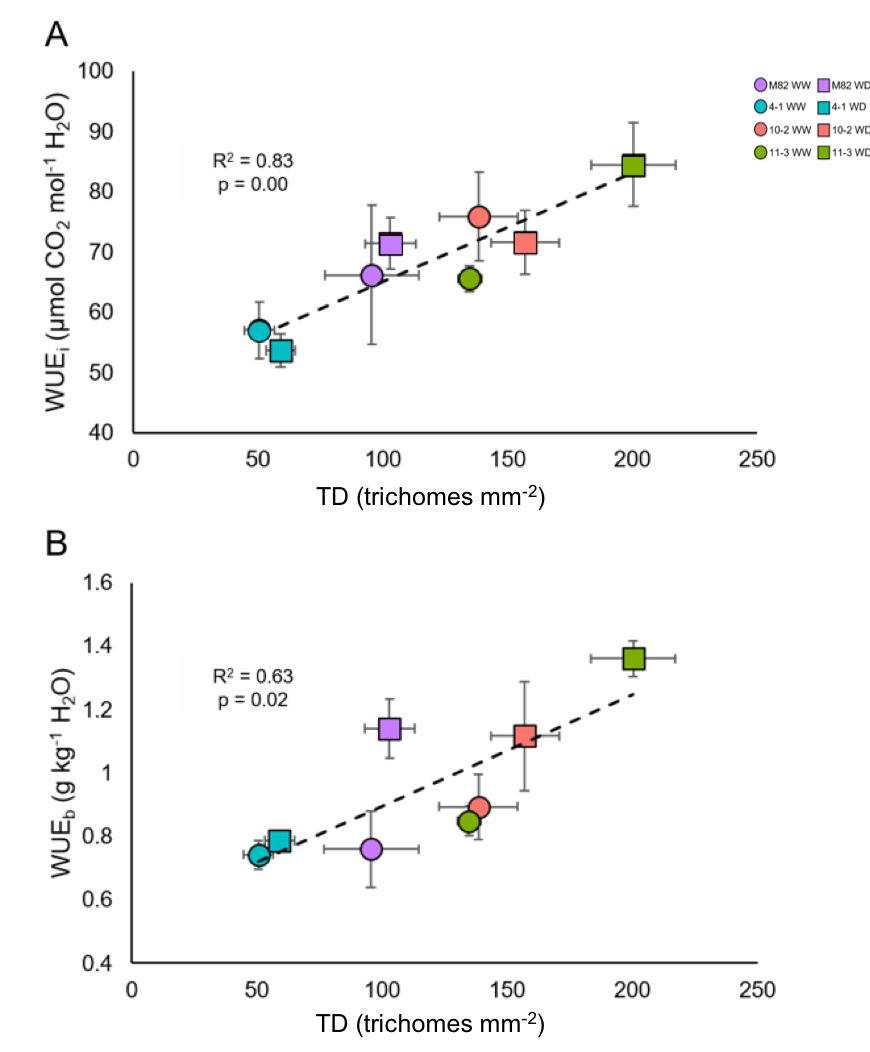


**Figure S7. Correlations between trichome density expressed per area unit and water use in lines M82, 4-1, 10-2 and 11-3 under WW and WD conditions.** (A) The correlation between TD and intrinsic water use efficiency (WUE_i_) (B) The correlation between TD and the plant-level water use efficiency (WUE_b_). Correlation coefficients and p-values by Pearson tests are shown in each graph. Purple markers represent M82, turquoise markers represent IL 4-1, red markers represent IL 10-2 and green markers represent IL 11-3. Circles represent WW values and squares represent WD values of field-grown plants. Values are means ± SE (n=4).


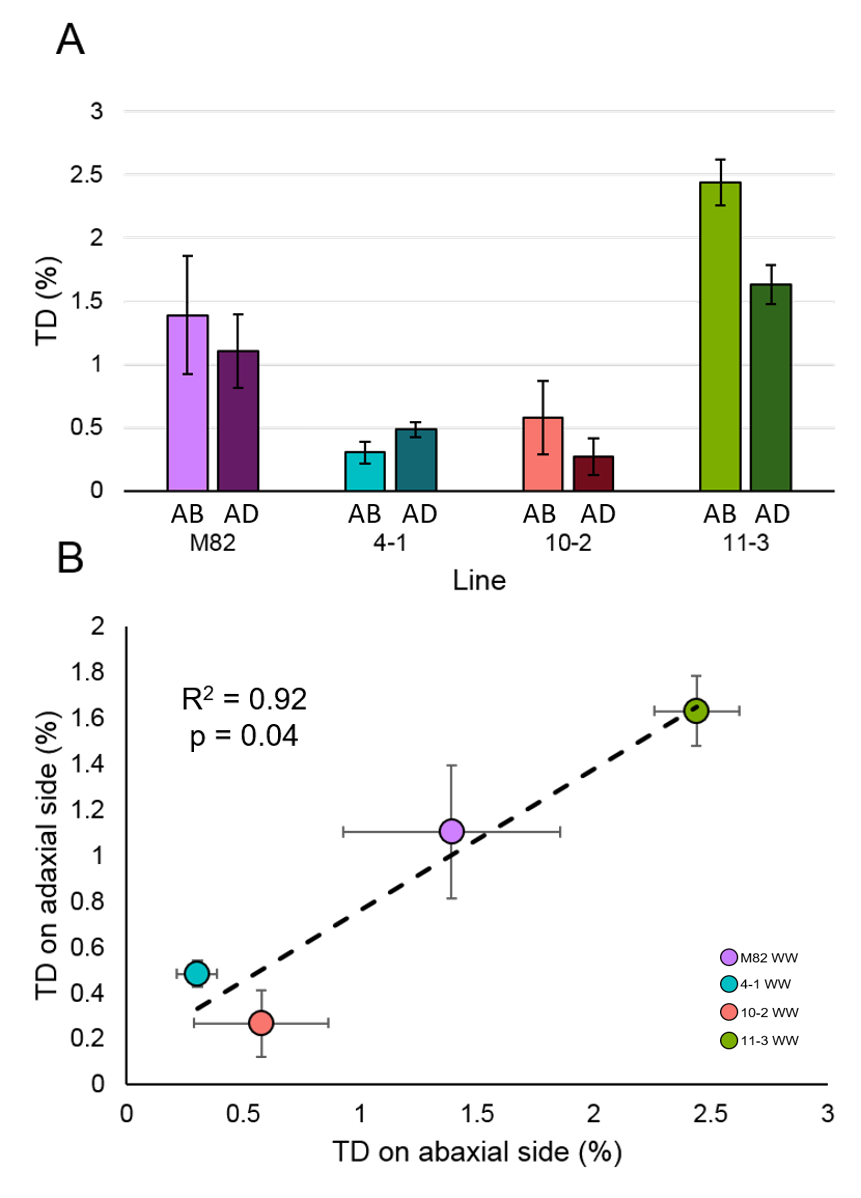


**Figure S8. Trichome densities on abaxial and adaxial sides of leaves of M82, IL4-1, IL10-2 and IL11-3 grown under greenhouse conditions.** (A) Trichome density for the abaxial (light colours, labelled AB) and adaxial (dark colours, labelled AD) sides of leaves are shown for M82 (purple), IL4-1 (turquoise), IL10-2 (pink) and IL11-3 (green) plants grown under greenhouse conditions (n=3). (B) The correlation between trichome density on the abaxial and adaxial sides is shown, colour-coded as in A. The Pearson correlation index is shown in the graph.


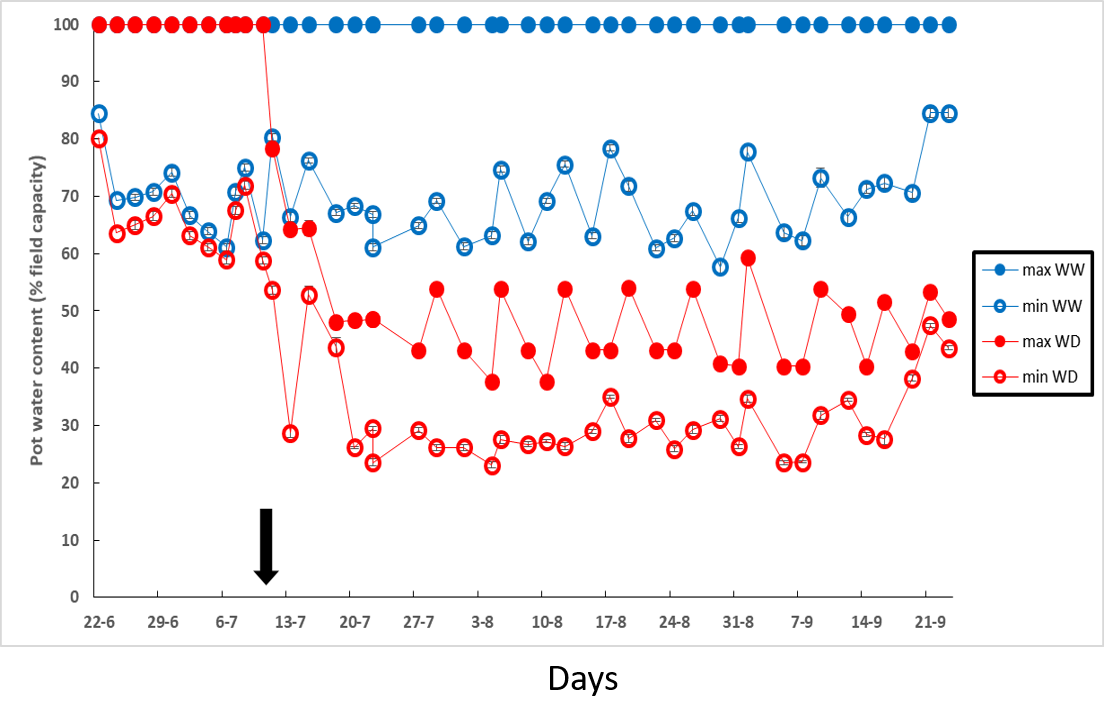


**Figure S9. Evolution of the pot water content during the experiment for the WW (blue) and WD (red) plants.** Empty dots correspond to the minimum value (i.e., just before irrigation) and solid dots represent the maximum value (i.e., just after irrigation) after treatment establishment. The arrow indicates when the treatment application started (11th July). All values are averages ± S.E. considering all the plants for each treatment.
