# Supplementary material for: The ratio of trichomes to stomata is associated with water use efficiency in Solanum lycopersicum (tomato)
Source: Plant J. 2018 Sep 5;96(3):607–19. doi: 10.1111/tpj.14055 (PMC6321981; doi:10.1111/tpj.14055)
Supplement: Supplementary file 2 — Table S1. Leaf morphological traits and photosynthetic characterization of the lines M82, 4‐1, 10‐2 and 11‐3 under field conditions before the onset of the drought treatment. Table S2. Dry biomass and total water supplied to plants upon completion of the experiment for lines M82, 4‐1, 10‐2 and 11‐3. [file TPJ-96-607-s002.docx]

**Supporting information**

|  | A_N_ | C 24h | g_s_ | g_m_ | g_tot_ | V_cmax_ | WUE_i_ |
| --- | --- | --- | --- | --- | --- | --- | --- |
| Acc. | μmol CO_2_ m^-2^s^-1^ | mol CO_2_ m^-2^ day^-1^ | mol H_2_O m^-2^s^-1^ | mol CO_2_ m^-2^s^-1^ | mol CO_2_ m^-2^ s^-1^ | μmol CO_2_ m^-2^s^-1^ | μmol CO_2_ mol^-1^ H_2_O |
| M-82 | 17.1 ± 1.16 ^a^ | 0.45 ± 0.03 ^a^ | 0.29 ± 0.046 ^a^ | 0.10 ± 0.015 ^a^ | 0.075 ± 0.016 ^a^ | 262 ± 4.26 ^a^ | 58.04 ± 3.70 ^a^ |
| 4-1 | 19.7 ± 1.35 ^a^ | 0.46 ± 0.07 ^a^ | 0.43 ± 0.027 ^a^ | 0.13 ± 0.004 ^a^ | 0.107 ± 0.013 ^a^ | 240 ± 2.76 ^a^ | 46.34 ± 2.68 ^a^ |
| 10-2 | 19.4 ± 1.26 ^a^ | 0.42 ± 0.04 ^a^ | 0.30 ± 0.026 ^a^ | 0.13 ± 0.015 ^a^ | 0.088 ± 0.006 ^a^ | 313 ± 5.29 ^a^ | 66.22 ± 7.09 ^a^ |
| 11-3 | 22.1 ± 1.43 ^a^ | 0.49 ± 0.04 ^a^ | 0.35 ± 0.026 ^a^ | 0.14 ± 0.017 ^a^ | 0.102 ± 0.004 ^a^ | 363 ± 6.92 ^a^ | 64.64 ± 4.56 ^a^ |

**Table S1. Leaf morphological traits and photosynthetic characterization of the lines M82, 4-1, 10-2 and 11-3 under field conditions before the onset of the drought treatment**. Net CO_2_ assimilation rate (A_N_), daily carbon fixation rate (C 24h) stomatal conductance (g_s_), mesophyll conductance (g_m_), total carbon conductance (g_tot_), maximum velocity of Rubisco carboxylation (V_cmax_) and intrinsic water use efficiency (WUE*_i_*) are expressed as mean±SE of five measurements per line. Analysis of variance (ANOVA) was used to test for differences between lines. Different letters represent statistically significant differences according to Tukey test (*P*<0.05).

|  | WW | | WD | |
| --- | --- | --- | --- | --- |
|  | Dry biomass (g) | Total water supplied (kg) | Dry biomass  (g) | Total water supplied (kg) |
| M-82 | 75.1 ± 6.5^a^ | 52.3 ± 1.8^ab^ | 80.5 ± 4.4^ab^ | 39.4 ± 0.6^a*^ |
| 4-1 | 78.0 ± 3.2^a^ | 56.4 ± 1.0^a^ | 65.4 ± 1.1^b*^ | 40.7 ± 0.9^a*^ |
| 10-2 | 92.0 ± 5.5^a^ | 54.7 ± 1.8^ab^ | 90.1 ± 10.5^a^ | 40.7 ± 2.7^a*^ |
| 11-3 | 79.3 ± 3.3^a^ | 49.6 ± 1.2^b^ | 85.7 ± 2.0^ab^ | 35.8 ± 1.0^a*^ |

**Table S2. Dry biomass and total water supplied to plants upon experiment completion for lines M82, IL 4-1, IL 10-2 and IL 11-3.** Dry biomass and total water supplied are expressed as mean±SE of four plants per line and treatment. Analysis of variance (ANOVA) was used to test for differences between lines. Different letters represent statistically significant differences among accessions according to Tukey test (*P*<0.05) within each treatment. Asterisks indicate significant differences between treatments for each line.
